# Influence of ethnicity on acceptability of method of blood pressure monitoring: a cross-sectional study in primary care

**DOI:** 10.3399/bjgp16X685717

**Published:** 2016-06-07

**Authors:** Sally Wood, Sheila M Greenfield, M Sayeed Haque, Una Martin, Paramjit S Gill, Jonathan Mant, Mohammed A Mohammed, Gurdip Heer, Amanpreet Johal, Ramandeep Kaur, Claire Schwartz, Richard J McManus

**Affiliations:** Nuffield Department of Primary Care Health Sciences, NIHR School for Primary Care Research, University of Oxford, Oxford.; Institute of Applied Health Research, University of Birmingham, Birmingham.; Institute of Clinical Sciences, College of Medical and Dental Sciences, University of Birmingham, Birmingham.; Institute of Clinical Sciences, College of Medical and Dental Sciences, University of Birmingham, Birmingham.; Institute of Applied Health Research, University of Birmingham, Birmingham.; Primary Care Unit, University of Cambridge, Cambridge.; Faculty of Health Studies, University of Bradford, Bradford.; Institute of Applied Health Research, University of Birmingham, Birmingham.; Institute of Applied Health Research, University of Birmingham, Birmingham.; Institute of Applied Health Research, University of Birmingham, Birmingham.; Nuffield Department of Primary Care Health Sciences, NIHR School for Primary Care Research, University of Oxford, Oxford.; Nuffield Department of Primary Care Health Sciences, NIHR School for Primary Care Research, University of Oxford, Oxford.

**Keywords:** blood pressure monitoring, ethnicity, hypertension, patient satisfaction, primary care

## Abstract

**Background:**

Ambulatory and/or home monitoring are recommended in the UK and the US for the diagnosis of hypertension but little is known about their acceptability.

**Aim:**

To determine the acceptability of different methods of measuring blood pressure to people from different minority ethnic groups.

**Design and setting:**

Cross-sectional study with focus groups in primary care in the West Midlands.

**Method:**

People of different ethnicities with and without hypertension were assessed for acceptability of clinic, home, and ambulatory blood pressure measurement using completion rate, questionnaire, and focus groups.

**Results:**

A total of 770 participants were included, who were white British (*n* = 300), South Asian (*n* = 241), and African Caribbean (*n* = 229). White British participants had significantly higher successful completion rates across all monitoring modalities compared with the other ethnic groups, especially for ambulatory monitoring: white British (*n* = 277, 92% [95% confidence interval [CI] = 89% to 95%]) versus South Asian (*n* = 171, 71% [95% CI = 65% to 76%], *P*<0.001) and African Caribbean (*n* = 188, 82% [95% CI = 77% to 87%], *P*<0.001), respectively. There were significantly lower acceptability scores for minority ethnic participants across all monitoring methods compared with white British participants. Focus group results highlighted self-monitoring as most acceptable and ambulatory monitoring least acceptable without consistent differences by ethnicity. Clinic monitoring was seen as inconvenient and anxiety provoking but with the advantage of immediate professional input.

**Conclusion:**

Reduced acceptability and completion rates among minority ethnic groups raise important questions for the implementation and interpretation of blood pressure monitoring. Selection of method of blood pressure monitoring should take into account clinical need, patient preference, and potential cultural barriers to monitoring.

## INTRODUCTION

High blood pressure is a key risk factor for the development of cardiovascular disease.^1^ Blood pressure has traditionally been measured in the clinic setting; however, data suggest that out-of-office measurement, particularly ambulatory blood pressure monitoring (ABPM), is more accurate in diagnosing hypertension.^2^^,^^3^ These findings have been incorporated into recent international clinical guidelines for the diagnosis of hypertension.^4^^–^7

Use of out-of-office blood pressure monitoring is likely to be strongly influenced by acceptability to patients. This may vary with both lifestyle and culture, hence the potential impact of ethnicity, which is also associated with cardiovascular prognosis.^8^^,^^9^

There have been few studies concerning the acceptability of different methods of blood pressure monitoring. Those that have been undertaken suggest that ABPM is associated with discomfort and sleep disturbance, although physicians may be able to make better treatment decisions as a result.^10^^–^12 A Greek study found that 62% considered ABPM more reliable than home monitoring but that 60% would choose home monitoring for their next evaluation.^13^ No studies explicitly considered the role of ethnicity in the determination of patient preferences, nor presented results in the light of likelihood of completion of a particular method. This mixed methods study aimed to ascertain acceptability of different methods of blood pressure monitoring to people from different minority ethnic groups and to develop an understanding of the factors underpinning their preferences.

## METHOD

### Participants

This work formed part of the Blood Pressure in Ethnic Groups Study (BP-Eth) for which detailed methods, including for the qualitative work, have been described previously.^14^^,^^15^ In brief, people of white British, South Asian, and African Caribbean ethnicities were recruited via their GP to have their blood pressure measured by different methods in 2010–2012. It was originally intended to recruit people of white Irish ethnicity but this proved not to be possible. Participants were purposefully sampled on the basis of both ethnicity and hypertension status from those responding to an initial survey and agreeing to take part in further research.^14^

### Procedures

Participants attended three research clinic appointments and had their blood pressure measured on each occasion. Between clinic visits their home blood pressure was measured for 1 week, and ambulatory blood pressure was measured for 24 hours.^14^^,^^16^^,^^17^

**How this fits in**

Ambulatory and home blood pressure monitoring are now recommended in both the UK and the US but little is known about the acceptability of these methods, particularly among minority ethnic groups. This research has shown that home monitoring, and particularly ambulatory monitoring, are less likely to be completed by minority ethnic individuals, even in a research setting with multilingual facilitators. Acceptability of ambulatory monitoring as measured by questionnaire and in qualitative focus groups was lower than either home or clinic measurement. Clinicians’ decisions regarding method of blood pressure monitoring should take into account both clinical need and patient preference, particularly for those from minority ethnic populations.

Completion rates for each method were defined using standard definitions as follows:^18^

- recording of clinic blood pressure at each of the three clinic appointments;
- 12 home readings on at least 4 days in the measurement week; and
- at least 14 valid daytime ambulatory blood pressure readings.

Previously validated acceptability questionnaires were completed following each method (first occasion for clinic readings).^11^

A convenience sample of participants willing to take part in an embedded focus group study was purposefully chosen to represent males and females as well as the three ethnic groups (that is, six groups, one for males and females of each ethnicity).^19^^,^^20^ Experiences and views of all three blood pressure measurement methods both within the study and in their general experience were discussed. The topic guide is included as Appendix 1. Focus groups were undertaken contemporaneously and independently before the quantitative analysis was complete. Data saturation was not sought because the aim was to gain information to explain and extend the quantitative findings.

### Analysis

Outcome data for the quantitative analysis comprised:

- completion rates for each method;
- acceptability using a previously validated questionnaire; and^11^
- a rank order of preference for each method. This included both doctor- and nurse-measured clinic blood pressure in order to be comparable with other studies using the same ranking system.

A three-level hierarchical model was developed: level 1 acceptability score, level 2 patient, and level 3 general practice. The model had a pre-specified set of covariates: ethnicity, age, sex, marital status, Index of Multiple Deprivation (IMD 2007),^21^ employment status, body mass index (BMI), smoking status, alcohol consumption, cholesterol, cardiovascular disease, chronic kidney disease, diabetes, and hypertension. The study hypothesis was addressed by a two-way interaction term between method of measurement and ethnicity. All analyses were undertaken using Stata (version 12).

The study was powered on the ability to detect differences in blood pressure between the different ethnic groups rather than acceptability, but retained the power of 80% at a 5% two-sided significance to detect a 10% difference in completion rates between ethnic groups assuming there were at least 219 participants in each group and that the rate was 80% on one group and 70% in the other.

Focus group transcripts were analysed thematically then triangulated and coded.^22^ Themes relating to the acceptability of the three modalities were extracted and a framework developed showing how they related to each other. Methodological triangulation was undertaken comparing the focus group results with those from the quantitative analysis.^23^

## RESULTS

### Participant characteristics

Data were available from 770 participants, (481 [63%] known to be hypertensive) from the three ethnic groups under consideration (Table 1). One white British individual was excluded with no blood pressure readings or acceptability question results. Mean age was 59 years, 51% were female, and mean BMI was just <30 kg/m^2^.

**Table 1.** Demographic characteristics, completion rates, and acceptability scores

**Characteristic**	**Total, *n* (%) unless otherwise stated**	**White British, *n* (%) unless otherwise stated**	**South Asian, *n* (%) unless otherwise stated**	**African Caribbean, *n* (%) unless otherwise stated**
Total participants	770	300 (39)	241 (31)	229 (30)
Male sex	374 (49)	154 (51)	132 (55)	88 (38)
Mean age (SD)	59 (9.6)	62 (8.7)	56 (9.4)	57 (9.7)
Mean body mass index (SD)	29.7 (5.6)	30.1 (5.8)	28.4 (4.3)	30.5 (6.3)
Previous history of hypertension	481 (63)	184 (61)	144 (60)	153 (67)
Previous history of coronary heart disease or stroke	128 (17)	64 (21)	33 (14)	31 (14)
Successfully completed clinic monitoring (all three occasions)^a^	710 (92, 90 to 94)	287 (96, 93 to 98)	214 (89, 84 to 92)	209 (91, 87 to 94)
Successfully completed home monitoring (at least 12 readings)^a^	715 (93, 91 to 95)	292 (97, 95 to 99)	220 (91, 87 to 94)	203 (89, 84 to 92)
Successfully completed ambulatory monitoring (at least 14 daytime readings)^a^	636 (83, 80 to 85)	277 (92, 89 to 95)	171 (71, 65 to 76)	188 (82, 77 to 87)
**Mean adjusted acceptability scores^b^**				
Ambulatory monitoring	2.9 (2.8 to 3.0)	2.7 (2.6 to 2.9)	3.1 (2.9 to 3.2)	3.0 (2.8 to 3.1)
Clinic monitoring	2.4 (2.3 to 2.4)	2.2 (2.1 to 2.4)	2.4 (2.3 to 2.6)	2.5 (2.4 to 2.6)
Home monitoring	2.1 (2.0 to 2.2)	1.9 (1.8 to 2.1)	2.2 (2.1 to 2.4)	2.3 (2.1 to 2.4)

a 95% CIs given in each case.

b All participants provided acceptability scores based on the first day of clinic readings. Acceptability scores are mean (95% CI) and a composite of 13 items. Lower scores reflect better acceptability.

SD = standard deviation.

### Blood pressure monitoring completion rates

White British participants had the highest completion rates across all methods of measurement (Table 1). South Asian participants were significantly less likely to complete ABPM (*n* = 171, 71% [95% CI = 65% to 76%]) than either African Caribbean (*n* = 188, 82% [95% CI = 77% to 87%], *P* = 0.004) or white British (*n* = 277, 92% [95% CI = 89% to 95%], *P*<0.001). Both minority ethnic groups were less likely to complete home monitoring than white British participants (Table 1).

### Acceptability

**Figure 1.**
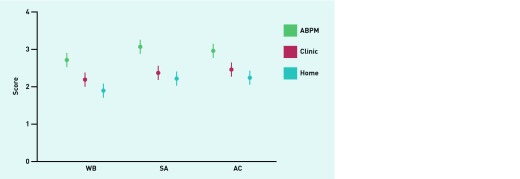
***Mean acceptability scores by ethnic group and method of blood pressure measurement (+/− 95% CIs). WB = white British, SA = South Asian, AC = African Caribbean.***

South Asian and African Caribbean groups gave lower acceptability for each method of blood pressure measurement than white British participants, but these were significant only for ABPM for South Asians compared with white British participants (Table 2, Figure 1). Overall, ambulatory monitoring was less acceptable than either clinic or home measurement, with differences in the questionnaire items regarding disturbances of activities, sleep, work, and general discomfort (Appendix 2, Figure 1).

**Table 2.** Mean acceptability scores of blood pressure monitoring method^a^

**Method**	**Type of score**	**White British mean (95% CI)**	**South Asian mean (95% CI)**	**African Caribbean mean (95% CI)**
**ABPM**	Raw	2.8 (2.7 to 2.9)	3.1 (2.9 to 3.2)	2.9 (2.8 to 3.1)
	Adjusted	2.7 (2.6 to 2.9)	3.1 (2.9 to 3.2)	3.0 (2.8 to 3.1)
**Clinic**	Raw	2.3 (2.2 to 2.4)	2.4 (2.3 to 2.5)	2.5 (2.4 to 2.6)
	Adjusted	2.2 (2.1 to 2.4)	2.4 (2.3 to 2.6)	2.5 (2.4 to 2.6)
**Self**	Raw	2.0 (1.9 to 2.1)	2.2 (2.1 to 2.3)	2.3 (2.2 to 2.4)
	Adjusted	1.9 (1.8 to 2.1)	2.2 (2.1 to 2.4)	2.3 (2.1 to 2.4)

a Scores are composite of 13 items (see Appendix 2 for individual scores). Lower scores reflect better acceptability.

ABPM = ambulatory blood pressure monitoring.

### Ranking

Ranking by method of measurement also showed ambulatory monitoring to be significantly less popular than the other methods. Self-monitoring was ranked highest with a small but significant difference over clinic measurements (Table 3). There was little difference in the order by which methods were ranked between ethnic groups.

**Table 3.** Preference ranking of method of blood pressure monitoring^a^

**Measurement**	**ABPM**	**Self**	**Nurse^b^**	**Doctor^b^**
**Mean (95% CI)**	0.5 (0.5 to 0.6)	2.2 (2.1 to 2.3)	1.8 (1.8 to 1.9)	1.5 (1.4 to 1.6)
**Median (IQR)**	0.0 (0.0 to 0.0)	3.0 (3.0 to 3.0)	2.0 (2.0 to 2.0)	1.0 (1.0 to 1.0)

a *Higher numbers indicate more favourable ranking.* P*<0.001 for each comparison (Friedman’s ANOVA followed by pairwise post-hoc test).*

b Patients were asked to rank clinic measurements by both nurse and doctor although this study only included measurements by a nurse/research facilitator.

ABPM = ambulatory blood pressure monitoring.

IQR = interquartile range.

### Focus group results

**Figure 2.**
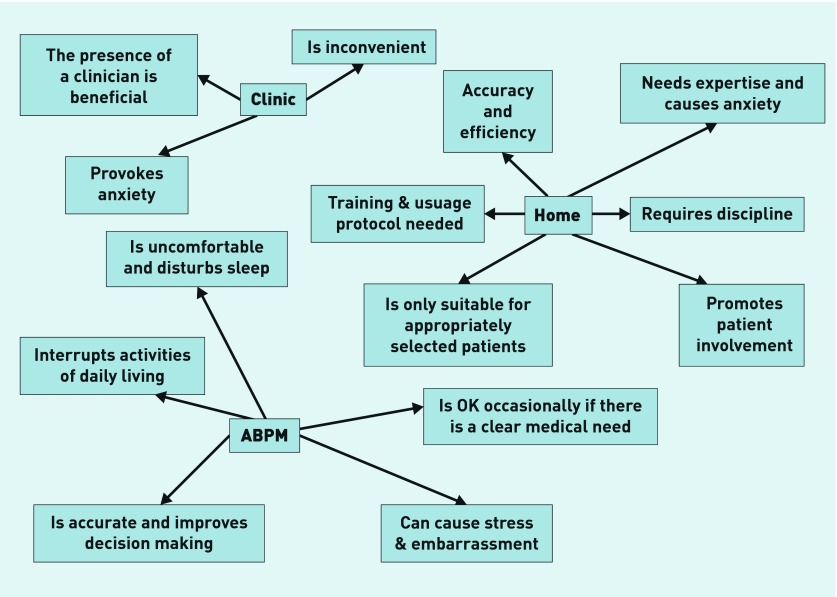
***Thematic representation of focus group results. ABPM = ambulatory blood pressure monitoring.***

The characteristics of the 37 focus group participants are summarised in Appendix 3. The overall thematic framework developed in the analysis linked emergent themes to each modality of measurement (Figure 2). Quotes relating to each theme are labelled by ethnic group, sex, and participant number.

### Office monitoring

The presence of a clinician, increased anxiety, and inconvenience were the key themes to emerge regarding the acceptability of office monitoring (Figure 2). Although similar numbers of statements regarding preferences for office monitoring were made in all of the focus groups, minority ethnic groups had more negative views.

#### Presence of a clinician

Participants from four out of six focus groups felt that a key benefit arising from office monitoring was the presence of a clinician while measurements were being made. This was due to a perceived improvement in the accuracy of readings resulting from a professional executing the process and their immediate interpretation of results, thus enabling any necessary action to be promptly taken:

‘… the purpose of the exercise was to get as accurate information as possible … so when it was done by the professional, well, I thought that was going to be perfect.’

(AC, M, 5)

‘Well, I think the fact that you’re in the right place and that you’re not the expert … and if there are any issues … well, at least there is some experience and expertise around.’

(SA, M, 4)

#### Anxiety and inconvenience

Every focus group apart from white British males mentioned anxiety caused by the office environment as an issue leading to falsely high readings. Some white British females found that the cuff that was used in the study for clinic readings sometimes caused bruising and members of the African Caribbean male group found attending the clinic inconvenient:

‘When I was taking it myself I was quite calm … but there’s something about coming up here that I don’t … I don’t cut it, I don’t like coming up to hospital and surgery so I get all wound up.’

(AC, F, 3)

‘The one you did yourself was much better, it saves you coming to the doctors … it’s much easier to do at home than coming in.’

(AC, M, 3)

### Home monitoring

Home monitoring was popular and preferred by all three ethnic groups, particularly African Caribbean participants. Key positive themes emerging were the ease of home monitoring, its accuracy and/or efficiency, and increased patient involvement. Conversely, some expressed concern about the need for timing and discipline whereas others doubted their own competence in executing the method.

#### Ease of home monitoring

All six focus groups reported that home monitoring was straightforward both in terms of executing the process and fitting it around daily activities. The two South Asian groups in particular found it very convenient:

‘I mean, taking it, is a doddle. It’s extremely easy to do when you know what you’re doing.’

(WB, F, 7)

#### Accuracy and efficiency

Self-monitoring was also seen as offering improved efficiency over other methods due to the increased number of readings resulting from a relatively low input of time. This was considered to improve accuracy, as was the ‘relaxing’ nature of the home setting, which was felt to enable a better representation of blood pressure:

‘I think that the GP should be able to decide better about the medication because I think that when you are at home you are more calm and relaxed, so your blood pressure reading should be alright.’

(SA, F, 1)

#### Increased patient involvement

Self-monitoring was considered to promote patient involvement in the management of blood pressure:

‘I didn’t mind having it done, in fact I started to get more interested in my blood pressure.’

(AC, F, 6)

However, the white British female group felt that they needed explicit ‘permission’ from their doctor to self-monitor:

‘Doctors don’t like people taking their blood pressure all the time … and I do think that there is resistance within doctors to people to keep on taking their own blood pressure … there is that idea that “leave it to the experts”.’

(WB, F, 1)

#### Timing and discipline

Remembering to home monitor was an issue raised by all ethnic groups, particularly for those doing shift work. This related to the guideline stipulation to take both morning and evening measurements between 6 and 12 o’clock:^24^

‘… at home because of the time limit that we were given, when we could take the morning and the evening, being a part-time worker, working shift work, I was very limited … to when I could do mine.’

(WB, F, 6)

#### Anxiety and expertise

There were concerns within all ethnic groups regarding accuracy of the equipment and lack of experience in executing the method:

‘… the question when you’ve got your own monitor, is, is it as good as the one that the GP’s got? You think … well, am I doing it right?’

(WB, F, 3)

Others felt that home monitoring was anxiety provoking due to the fear of a high reading and its associated health implications:

‘But when I got home and I thought, oh, you know, I’ve got to take my blood pressure I suddenly, I had a huge panic attack ... and then of course, when I took it, it was high ... I mean it was bound to be, wasn’t it?’

(WB, F, 7)

‘The first one I had was 206 mmHg — I thought I was going to die.’

(AC, M, 6)

### Ambulatory monitoring

ABPM was valued for its accuracy by all ethnic groups. However, this was tempered by its impact on daily activities and sleep, along with the embarrassment caused by others being aware of its presence.

#### Accurate and influences decision making

Ambulatory monitoring was widely seen to improve accuracy, thereby enabling better clinical decisions to be made about blood pressure:

‘I think that coming to your GP etc. isn’t a problem: but they’re just random snapshots so I’m more convinced about the 24-hour one taking an average.’

(WB, M, 4)

However, although only a minority supported its use on multiple occasions, a number of participants said that they were happy to do it as a ‘one-off’:

‘If we had to do it on a regular basis then I would find that really uncomfortable … but for the one day I didn’t mind.’

(AC, F, 4)

#### Influence of daily activities

White British participants particularly commented that ABPM measurements depended on what they had been doing on the measurement day. If this was not typical of their usual routine then some thought that the resulting readings might not represent their ‘true’ blood pressure:

*‘… but at the time I’d only just been made redundant so it was just a case of saying “this really isn’t a normal day” … so it would be interesting to see what the results would be* (if I had been more active).’

(WB, M, 4)

#### Disruption of sleep and other activities

More than twice as many negative as positive comments were made regarding ambulatory monitoring. A key issue here was disruption to sleep and other activities due to discomfort. Such views were held regardless of ethnicity:

*‘I didn’t get much sleep* (on the day of the ABPM) *because as soon as it started it woke me up.’*

(AC, F, 3)

#### Embarrassment, medicalisation, and anxiety

The fact that monitoring occurred throughout the day and was obvious to others, hence resulting in potential embarrassment, were particular issues reported by South Asian and African Caribbean participants:

‘If the design was a bit more discreet and a little bit more user-friendly then maybe we would have had a different experience but as it stands now, you know, it is intrusive.’

(SA, M, 4)

‘… what I did mind was walking along the road and then I would get the warning and have to stop … and people were watching me … and it was so embarrassing.’

(AC, F, 6)

Both minority ethnic groups commented on the anxiety that ambulatory monitoring brought on and the impression given to others of having a medical problem:

‘… ‘cos I live with my in-laws I had to hide it from them ‘cos I didn’t want them to get worried that there was something wrong.’

(SA, F, 5)

## DISCUSSION

### Summary

This study has evaluated acceptability data from a large group of people drawn from white British and two major minority ethnic groups, and has shown that, although all methods of blood pressure monitoring were broadly acceptable to people from all three ethnic groups, ambulatory monitoring was less favoured. Furthermore, South Asian and African Caribbean participants found all types of monitoring significantly less acceptable than those from the white British group and this was reflected in lower completion rates, particularly for South Asian participants. Given UK and international guidelines on the use of ambulatory monitoring for the diagnosis of hypertension, a 20% difference in completion of such monitoring could have a significant impact on the quality of care across ethnic communities.^6^^,^^25^ Conversely, self-monitoring proved popular with all participants. The consistency of results across quantitative and qualitative methodologies suggests that genuine differences exist in acceptability between methods and between ethnic groups.

### Strengths and limitations

To the authors’ knowledge, this is the first study to gain detailed information on the acceptability and performance of different methods of blood pressure monitoring in a large multiethnic population. This is important because such monitoring is such a common aspect of clinical management, particularly in primary care. The results are strengthened by using a combination of methods.

Participants were recruited from one area of the UK (the West Midlands), and homogeneity within ethnic groups has been assumed. This might potentially limit generalisability in that there may be differences within the ethnic categories used in this study. However, the uniformity of responses from multiple methods by those of different ethnic groups suggests that this is unlikely to have affected the headline results.

Recruitment relied on purposive sampling of a pool of volunteers to ensure that all three minority ethnic groups were represented, as were those with and without a diagnosis of hypertension. Responders from minority ethnic groups were younger and this was taken into account in the statistical analysis.^26^ More participants had a previous diagnosis of hypertension than not, although this might be expected to lead to better rather than worse acceptability given prior exposure.

### Comparison with existing literature

In common with previous studies, this work has shown that ambulatory monitoring is less acceptable than other methods of blood pressure measurement.^11^^–^13 Compared with previous work in the UK, Greece, and in a largely white area of the US, the current study has extended these findings to African Caribbean and South Asian groups. To the authors’ knowledge, the reduced acceptability of ABPM by minority ethnic groups, and particularly South Asians, has not been reported before.

Although relative preferences for modality of blood pressure monitoring were broadly consistent between ethnic groups, South Asian and African Caribbean participants rated all modalities of blood pressure monitoring less favourably than did their white British counterparts, albeit only approaching significance for the South Asian group for ABPM. This fits with data suggesting that minority ethnic groups rate various aspects of primary care less favourably than white British groups.^27^

Responders considered ABPM to be acceptable when there was a clear medical need, as it resulted in improved accuracy, reinforcing findings from the US and Greece.^12^^,^^13^ The most commonly reported issue with ABPM in the focus groups and questionnaire responses was disturbance of sleep, hence the use of daytime ambulatory monitoring alone might improve this.

The positive views on home monitoring expressed here reinforce similar findings from previous trials.^28^^,^^29^ However, this method has been shown to have only moderate diagnostic agreement when tested against the reference of ABPM.^2^ Nonetheless, longitudinal studies have shown improved prognostic power with home readings compared with clinic readings and recent Japanese guidelines have incorporated self-monitoring for both diagnosis and ongoing management.^30^^,^^31^

### Implications for practice

Around 20% fewer South Asian individuals completed the minimum acceptable number of ambulatory measurements than white British people, despite a multilingual research team, availability of translated research materials, and probably longer explanations than may occur in daily practice. This seems from the focus groups to have been at least in part because of issues of embarrassment compounded by questions from extended family that may be more relevant to those from minority ethnic groups. Serious consideration as to how this problem can be addressed is needed if the benefits of accurate monitoring and, particularly, diagnosis are to be extended to all. An important issue is the current often bulky and noisy ambulatory monitoring technology. New methods of indirect blood pressure monitoring that do not require inflating cuffs are under development and may address this.^32^

Greater use of home monitoring in the management of hypertension seems likely to be supported by people of all ethnicities. In the meantime, clinic monitoring currently retains a significant role in the management of hypertension despite its inaccuracy.^5^^,^^25^ Clinicians’ decisions regarding the method of blood pressure monitoring for an individual should take into account both clinical need and patient preference. However, a discussion of lifestyle and cultural factors, particularly with those from minority ethnic groups, may be required to maximise the quality of care provided.^33^ In blood pressure monitoring, ethnicity is relevant.

## Appendix 1. Topic guide for focus groups

### Office measurements

- How did you find the experience of having your blood pressure measured in the clinic?
- How did this process make you feel?
- Do you think that having your blood pressure taken at the clinic is an accurate way of measuring it?
- How convenient is it for you to have your blood pressure measured at the clinic?

### Home monitoring

- Prior to this study, had any of you taken your blood pressure with a home monitor? If so, had you had any training on this?
- How was the experience of home monitoring in this study for you?
- How did knowing the readings make you feel (particularly if they were either high or low)?
- How easy was it to monitor your blood pressure at home? Which factors may have made it more difficult (for example, machine problems, fitting this in with normal daily routines)?
- Following this experience, are you interested in continuing to monitor your blood pressure at home? Would you consider buying your own machine? Why/not? How would you feel about passing these readings on to your GP?
- Would you be prepared to measure your blood pressure at home if it meant that you didn’t need to have your blood pressure measured at the GP surgery?
- Do you think that the readings obtained through home monitoring will affect the way that your GP manages your blood pressure? If so, how?

### Ambulatory monitoring

- How was the experience of wearing the ambulatory cuff for 24 hours (what did you like/not like)?
- What impact did it have on your daily activities, for example, washing/driving/work/sleep or personal relationships? As a result, did you take the cuff off at all?
- Did you experience any technical problems (for example, with the machine)?
- Would you be prepared to wear the cuff for 24 hours once a year, if it meant that you didn’t need to have your blood pressure measured at the GP surgery?
- Do you think that the readings obtained through ambulatory monitoring will affect the way that your GP manages your blood pressure? If so, how?

### Results

*Introductory statement that all results are confidential and that there is no need to discuss further if the patient isn’t comfortable.*

- How did you feel about getting your results?
- If there was any significant difference between methods, why do you think that was?
- Was this information useful to you? Were the results what you were expecting?
- Has this led to any changes in the way that your blood pressure is managed?

### Concluding

- Of the three methods that were trialled, which would you prefer to use in the future? Why?
- Which method would you least like to use in the future? Why?
- Is there anything else that you’d like to tell us?

## Appendix 2. Acceptability of different methods of blood pressure monitoring by ethnicity, *n* = number completing the relevant questionnaire

**Table:**

**Statement^a^**	**Clinic**				**ABPM**				**Home**			
	**All (*n* = 769) Med (IQR)**	**White British (*n* = 300) Med (IQR)**	**South Asian (*n* = 240) Med (IQR)**	**African Caribbean (*n* = 229) Med (IQR)**	**All (*n* = 715) Med (IQR)**	**White British (*n* = 292) Med (IQR)**	**South Asian (*n* = 213) Med (IQR)**	**African Caribbean (*n* = 210) Med (IQR)**	**All (*n* = 727) Med (IQR)**	**White British (*n* = 293) Med (IQR)**	**South Asian (*n* = 223) Med (IQR)**	**African Caribbean (*n* = 211) Med (IQR)**
It made me anxious	2 (1–5)	2 (1–3)	2 (1–4.5)	2 (2–5)	2 (1–5)	2 (1–3)	2 (1–5)	2 (2–5)	2 (1–2)	2 (1–2)	2 (1–5)	2 (1–5)
It disturbed activities	2 (1–3)	2 (1–3)	2 (1–4)	2 (1–2)	5^b^ (2–6)	5^b^ (2–6)	5^b^ (2–6)	5^b^ (2–6)	2 (1–2)	2 (1–2)	2 (1–2)	2 (1–2)
It disturbed sleep	2 (1–4)	2 (1–4)	2 (1–4)	2 (1–4)	3^b^ (2–6)	3^b^ (2–6)	5^b^ (2–7)	2 (2–6)	2 (1–2)	2 (1–2)	1 (1–2)	2 (1–2)
It disturbed work	2 (1–4)	2 (2–4)	2 (1–4)	2 (2–4)	4^b^ (2–6)	4^b^ (2–5)	4^b^ (2–6)	3 (2–6)	2 (1–2)	2 (1–2)	2 (1–2)	2 (1–2)
It was uncomfortable	2 (1–4)	2 (1–5)	2 (1–5)	2 (1–2)	5^b^ (2–6)	5^b^ (2–5)	5^b^ (2–6)	5^b^ (2–6)	2 (1–2)	2 (1–2)	2 (1–2)	2 (1–2)
I felt self-conscious	2 (1–2)	2 (1–2)	2 (1–2)	2 (2–5)	2 (2–5)	2 (2–3)	2 (2–6)	2 (2–6)	2 (1–2)	2 (1–2)	2 (1–2)	2 (1–3)
I was unsure what to do	2 (1–2)	2 (1–2)	2 (1–2)	2 (1–2)	2 (1–2)	2 (1–2)	2 (1–2)	2 (1–2)	2 (1–2)	2 (1–2)	1 (1–2)	2 (1–2)
There was a lot of waiting around	2 (1–2)	2 (1–2)	2 (1–2)	2 (1–2)	2 (1–2)	2 (1–2)	2 (1–3)	2 (2–2)	2 (1–2)	2 (1–2)	2 (1–2)	2 (1–2)
It worried me, knowing the blood pressure	2 (1–3)	2 (1–2)	2 (1–5)	2 (2–5)	2 (1–3)	2 (1–2)	2 (1–4)	2 (2–3)	2 (1–5)	2 (1–3)	2 (1–5)	2 (2–5)
It was difficult to remember to do it	2 (2–4)	3 (2–4)	2 (1–4)	2 (2–4)	2 (1–4)	2 (1–4)	2 (1–4)	2 (2–2)	2 (1–2)	2 (1–2)	2 (1–5)	2 (1–3)
It is accurate^c^	2 (1–2)	2 (1–2)	2 (1–2)	2 (1–2)	2 (1–2)	2 (1–2)	2 (1–2)	2 (1–2)	2 (1–2)	2 (1–2)	2 (1–2)	2 (1–2)
I felt in control^c^	2 (1–2)	2 (1–2)	2 (1–3)	2 (2–4)	2 (1–2)	2 (1–2)	2 (1–2)	2 (1–3)	2 (1–2)	2 (1–2)	2 (1–2)	2 (1–2)
It is a good use of doctor or nurse time^c^	2 (1–2)	2 (1–2)	2 (1–2)	2 (1–2)	2 (1–2)	2 (1–2)	2 (1–2)	2 (1–2)	2 (1–2)	2 (1–2)	2 (1–2)	2 (1–2)
Adjusted mean acceptability score (95% CI)	2.4 (2.3–2.4)	2.2 (2.1–2.4)	2.4 (2.3–2.6)	2.5 (2.4–2.6)	2.9 (2.8–3.0)	2.7 (2.6–2.9)	3.1 (3.0–3.2)	3.0 (2.8–3.1)	2.1 (2.0–2.2)	1.9 (1.8–2.1)	2.2 (2.1–2.4)	2.3 (2.1–2.4)
Adjusted median acceptability score (IQR)	2.3 (2.1–2.7)	2.3 (2.0–2.5)	2.3 (2.0–2.7)	2.5 (2.1–2.8)	2.9 (2.6–3.2)	2.8 (2.6–3.0)	3.0 (2.7–3.4)	3.0 (2.6–3.2)	2.1 (1.8–2.4)	2.0 (1.8–2.2)	2.1 (1.8–2.5)	2.3 (1.9–2.6)

a Each statement rated via 7-point Likert scale. Ratings: 1 = disagree strongly; 2 = disagree; 3 = disagree slightly; 4 = unsure or not applicable; 5 = agree slightly; 6 = agree; 7 = agree strongly. Acceptability score is mean of all 13 individual questions.

b *Significant difference at* P*=0.05 for these items versus other methods of measurement.*

c Scoring reversed for positive items (accurate, control, good use of time), that is, 1 = agree strongly, 7 = disagree strongly.

IQR = interquartile range.

## Appendix 3. Focus group characteristics

**Table:**

**Variable**	**White British**		**South Asian**		**African Caribbean**	
	**Male**	**Female**	**Male**	**Female**	**Male**	**Female**
Order^a^	2	1	5	6	4	3
Number of participants	4	7	6	5	8	7
Median age years, (range)	71 (50–72)	67 (64–73)	63 (52–72)	46 (41–55)	55 (47–72)	64 (63–72)
Years in UK, median (range)	71 (50–72)	67 (64–73)	44 (28–48)	38 (25–41)	46 (5–52)	47 (44–48)
Hypertension, *n* (%)	2 (50)	4 (57)	4 (67)	2 (40)	5 (63)	7 (100)

a Order in which focus groups were held.

## References

[1] Lewington S, Clarke R, Qizilbash N (2002). Age-specific relevance of usual blood pressure to vascular mortality: a meta-analysis of individual data for one million adults in 61 prospective studies. Lancet.

[2] Hodgkinson J, Mant J, Martin U (2011). Relative effectiveness of clinic and home blood pressure monitoring compared with ambulatory blood pressure monitoring in diagnosis of hypertension: systematic review. BMJ.

[3] Roguin A (2006). Scipione Riva-Rocci and the men behind the mercury sphygmomanometer. Int J Clin Pract.

[4] Krause T, Lovibond K, Caulfield M (2011). Management of hypertension: summary of NICE guidance. BMJ.

[5] Mancia G, Fagard R, Narkiewicz K (2013). 2013 ESH/ESC Guidelines for the management of arterial hypertension. Blood Press.

[6] Daskalopoulou SS, Rabi DM, Zarnke KB (2015). The 2015 Canadian Hypertension Education Program recommendations for blood pressure measurement, diagnosis, assessment of risk, prevention, and treatment of hypertension. Can J Cardiol.

[7] Piper MA, Evans CV, Burda BU (2015). Diagnostic and predictive accuracy of blood pressure screening methods with consideration of rescreening intervals: a systematic review for the U.S. Preventive Services Task Force. Ann Intern Med.

[8] Gill PS, Kai J, Bhopal RS, Stevens A, Raftery J, Mant J, Simpson S (2007). Black and minority ethnic groups. Health care needs assessment: the epidemiologically based needs assessment reviews.

[9] Wild S, McKeigue P (1997). Cross sectional analysis of mortality by country of birth in England and Wales, 1970–92. BMJ.

[10] Mallion JM, de Gaudemaris R, Baguet JP (1996). Acceptability and tolerance of ambulatory blood pressure measurement in the hypertensive patient. Blood Press Monit.

[11] Little P, Barnett J, Barnsley L (2002). Comparison of acceptability of and preferences for different methods of measuring blood pressure in primary care. BMJ.

[12] Ernst ME, Bergus GR (2003). Favorable patient acceptance of ambulatory blood pressure monitoring in a primary care setting in the United States: a cross-sectional survey. BMC Fam Pract.

[13] Nasothimiou EG, Karpettas N, Dafni MG, Stergiou GS (2014). Patients’ preference for ambulatory versus home blood pressure monitoring. J Hum Hypertens.

[14] Wood S, Martin U, Gill P (2012). Blood pressure in different ethnic groups (BP-Eth): a mixed methods study. BMJ Open.

[15] Martin U, Haque MS, Wood S (2014). Ethnicity and differences between clinic and ambulatory blood pressure measurements. Am J Hypertens.

[16] Stergiou GS, Giovas PP, Gkinos CP, Patouras JD (2007). Validation of the Microlife WatchBP Home device for self home blood pressure measurement according to the International Protocol. Blood Press Monit.

[17] Mattu GS, Heran BS, Wright JM (2004). Overall accuracy of the BpTRU: an automated electronic blood pressure device. Blood Press Monit.

[18] Stergiou GS, Zourbaki AS, Skeva II, Mountokalakis TD (1998). White coat effect detected using self-monitoring of blood pressure at home: comparison with ambulatory blood pressure. Am J Hypertens.

[19] Patton MQ (2002). Qualitative research and evaluation methods.

[20] Teddlie C, Yu F (2007). Mixed methods sampling: a typology with examples. J Mix Methods Res.

[21] UK Government (2007). Indices of deprivation.

[22] Glaser B (1965). The constant comparative method of qualitative analysis. Soc Probl.

[23] Denzin NK (2006). Sociological methods: a sourcebook.

[24] Parati G, Stergiou GS, Asmar R (2010). European Society of Hypertension practice guidelines for home blood pressure monitoring. *J Hum Hyperten*s.

[25] National Institute for Health and Care Excellence (2011). *Hypertension in adults: diagnosis and management* CG127.

[26] Office for National Statistics. Focus on ethnicity and identity summary report, March 2005.

[27] Mead N, Roland M (2009). Understanding why some ethnic minority patients evaluate medical care more negatively than white patients: a cross sectional analysis of a routine patient survey in English general practices. BMJ.

[28] Jones MI, Greenfield SM, Bray EP (2012). Patients’ experiences of self-monitoring blood pressure and self-titration of medication: the TASMINH2 trial qualitative study. Br J Gen Pract.

[29] Bosworth HB, Olsen MK, Grubber JM (2009). Two self-management interventions to improve hypertension control: a randomized trial. Ann Intern Med.

[30] Ward AM, Takahashi O, Stevens R, Heneghan C (2012). Home measurement of blood pressure and cardiovascular disease: systematic review and meta-analysis of prospective studies. J Hypertens.

[31] Shimamoto K, Ando K, Fujita T (2014). The Japanese Society of Hypertension Guidelines for the Management of Hypertension (JSH 2014). Hypertens Res.

[32] Komori T, Eguchi K, Hoshide S (2013). Comparison of wrist-type and arm-type 24-h blood pressure monitoring devices for ambulatory use. Blood Press Monit.

[33] [No authors listed]. (1967). Effects of treatment on morbidity in hypertension. Results in patients with diastolic blood pressures averaging 115 through 129 mm Hg. JAMA.

